# De Novo Transcriptome of the Flagellate *Isochrysis galbana* Identifies Genes Involved in the Metabolism of Antiproliferative Metabolites

**DOI:** 10.3390/biology11050771

**Published:** 2022-05-18

**Authors:** Gennaro Riccio, Kevin A. Martinez, Adrianna Ianora, Chiara Lauritano

**Affiliations:** 1 Department of Ecosustainable Marine Biotechnology, Stazione Zoologica Anton Dohrn, Villa Comunale, 80121 Naples, Italy; gennaro.riccio@szn.it (G.R.); adrianna.ianora@szn.it (A.I.); 2 Fundación MEDINA, Centro de Excelencia en Investigación de Medicamentos Innovadores en Andalucía, Avda. del Conocimiento 34, 18016 Granada, Spain; kevin.martinez@medinaandalucia.es

**Keywords:** *Isochrysis galbana*, microalgae, transcriptome, transcriptomic-guided approach, drug discovery, antiproliferative activity, cancer cells

## Abstract

**Simple Summary:**

Microalgae are a huge renewable and eco-friendly source of natural compounds, including vitamins, carbohydrates, pigments, sterols and lipids. They have found applications in different industrial sectors, including the pharmaceutical, nutraceutical and cosmeceutical fields. *Isochrysis galbana*, a marine flagellate belonging to the Haptophyta phylum, thanks to its nutraceutical composition has been proposed as a shellfish diet (Shellfish Diet 1800^®^) as well as in the composition of cookies, fresh pasta and yogurt. *I. galbana* powder, extracts or pure molecules have shown interesting bioactivities, such as antioxidant, antidiabetes and antituberculosis, while the antiproliferative activity is mainly related to lung lymphoblasts. In the current study, we aimed to identify metabolic pathways activated in stressful conditions (stationary growth phase) by transcriptomic and bioactivity-guided fractionation. Overall, the results showed antiproliferative activities against melanoma cells, suggesting new possible applications in cancer prevention and treatment.

**Abstract:**

Haptophytes are important primary producers in the oceans, and among the phylum Haptophyta, the flagellate *Isochrysis galbana* has been found to be rich in high-value compounds, such as lipids, carotenoids and highly branched polysaccharides. In the present work, *I. galbana* was cultured and collected at both stationary and exponential growth phases. A transcriptomic approach was used to analyze the possible activation of metabolic pathways responsible for bioactive compound synthesis at the gene level. Differential expression analysis of samples collected at the exponential versus stationary growth phase allowed the identification of genes involved in the glycerophospholipid metabolic process, the sterol biosynthetic process, ADP-ribose diphosphatase activity and others. *I. galbana* raw extracts and fractions were tested on specific human cancer cells for possible antiproliferative activity. The most active fractions, without affecting normal cells, were fractions enriched in nucleosides (fraction B) and triglycerides (fraction E) for algae collected in the exponential growth phase and fraction E for stationary phase samples. Overall, transcriptomic and bioactivity data confirmed the activation of metabolic pathways involved in the synthesis of bioactive compounds giving new insights on possible *Isochrysis* applications in the anticancer sector.

## 1. Introduction

Microalgae are a huge renewable and eco-friendly source of natural compounds, including vitamins, carbohydrates, pigments, sterols and lipids, presenting several bioactivities for biotechnological applications in the pharmaceutical, nutraceutical and cosmeceutical sectors [1,2,3], such as antiviral [4], antimicrobial [5], anti-inflammatory [4], immunomodulatory [6] and anticancer activities [5]. Microalgae are found in both marine and terrestrial habitats due to their ability to adapt and survive in very different environments. They can also be cultivated on a large scale, both [7] indoors and outdoors [5], allowing high biomass and high yields of the metabolites of interest without negative impacts on the marine environment.

Haptophyta are one of the major components of the microbial ecosystem. They are distributed in both freshwater and marine ecosystems, mainly in tropical, temperate and polar oceans [8], and may form massive blooms, which can also be toxic [9]. Haptophyta include two classes: Prymnesiophyceae (Coccolitophyceae) and Pavlovophyceae [10]. They have as main pigments chlorophyll-a and -c, as well as β-carotene, fucoxanthin and other xanthophylls [10]. *Isochrysis galbana* (Isochrysidales) is a marine flagellate belonging to the Haptophyta phylum [11] and is one of the most studied microalga belonging to this phylum because it has been used and proposed for various applications. On the market, there is, for instance, a shellfish diet (Shellfish Diet 1800^®^) product based on five microalgae, including *Isochrysis*, (https://reedmariculture.com/products/shellfish-diet; accessed on the 21 March 2022), and various trials have been performed by including *I. galbana* in cookies, fresh pasta and yogurt (e.g., as omega-3 source; [10,12,13,14]) for human consumption.

*I. galbana* has been frequently used in aquaculture, mainly for mollusk larvae, fish and crustacean feeding in their early stages of growth because of its good nutritive characteristics [15,16]. *I. galbana* has been reported to be rich in lipids (e.g., PUFA), such as eicosapentaenoic acid (EPA) and docosahexaenoic acid (DHA) [17,18,19,20], known to be high-value molecules, with possible applications in the prevention of heart and circulatory diseases, as well as brain development. For instance, *I. galbana* has been shown to produce 1.08 mg^−1^ L^−1^ day^−1^ DHA when cultured at 14 °C [10].

It has a high content of carotenoids and, in particular, fucoxanthin, which can reach about 76% of the total carotenoids in this microalgal species [21]. In addition, *I. galbana* is rich in polysaccharides (contents can be up to 25% of dry cell weight [15]). It is known to be a good producer of exopolysaccharides used in aquaculture as fodder or feed supplements, as metal ion chelator and for plant protection against pathogens [10]. Sun and colleagues isolated three antioxidant polysaccharides, named IPSI-A, IPSI-B and IPSII, from *I. galbana* that showed scavenging activities against superoxide and hydroxyl radicals [15]. A highly branched (1→3,1→6)-β-D-glucan was isolated by Sadovskaya and coworkers [22] from *I. galbana* Parke from the Roscoff culture collection. The antiproliferative activity of the glucan was evaluated on lung lymphoblast U937 cells (ECACC) and results showed about 65% cell viability after 72 h of incubation in the presence of glucan at 100 μg·mL^−1^ [22]. The same authors also tested the immunomodulatory properties of the purified glucan by evaluating interleukin-8 expression by the human U937 monocytic cell line, but they did not observe any significant inhibitory or stimulatory effects. Finally, sterols, including 24-oxocholesterol acetate, ergost-5-en-3β-ol, cholest-5-en-24-1,3-(acetyloxy)-3β-ol and 24-methylcholesta-5,22-dien-3β-ol from *I. galbana* showed antituberculosis activity [23]. *I. galbana* was also tested in a diabetic rat model producing weight loss and decreased glucose, triacylglycerol and cholesterol values showing promising antidiabetic activities [24]. Altogether, these data showed that *I. galbana* can be the source of interesting bioactive compounds with various industrial applications from aquaculture to the food and pharmaceutical sectors.

Microalgal metabolite production may vary depending on the studied clones [25], growth phases [26], culturing conditions (the so-called OSMAC approach—one strain–many compounds [27,28]) and extraction method [29]. In the current study, we examined *I. galbana* CCMP1323 (from the Bigelow USA culture collection) in two different growth phases: the exponential phase, in which the microalgae have active cell division and the biomass usually increases exponentially, and the stationary phase, in which there is an equal rate of cell division and cell death, mainly due to depletion of nutrients in the medium [30]. A de novo transcriptome of *I. galbana* CCMP1323 was sequenced in the current study, and differential expression analyses were performed between algae collected at the exponential or stationary phase. The transcriptomic approach was used to identify variations in metabolic pathways that are potentially of interest. In addition, a bioactivity-guided fractionation was applied in order to screen for possible activities for cancer prevention/treatment. Tested fractions were enriched in metabolites whose metabolic pathways were differentially expressed as resulted by transcriptomic bioinformatics analyses. Four fractions, enriched in nucleosides, phospholipids and glycolipids, sterols and free fatty acids and triglycerides, respectively, were obtained by solid-phase extraction [31]. In this study, *I. galbana* CCMP1323 total extracts and fractions, coming from algae pelleted in the exponential or stationary phase, were screened for possible antiproliferative activity on different cancer cell lines, i.e., melanoma A2058, hepatocellular liver carcinoma HepG2 and lung adenocarcinoma A549, as well as a normal cell line (lung fibroblast MRC5 and human keratinocyte HaCat) used as a toxicity control. In the literature, various studies have reported that microalgae may produce bioactive secondary metabolites at the end of the stationary phase, when nutrients are limited and the algae are more stressed [32]. For this reason, we analyzed the antiproliferative activity of *I. galbana* at both the exponential and the stationary phase. This approach allowed the combination of transcriptomic pathway analysis and bioactivity screening in order to give new insights into possible applications of *I. galbana*.

## 2. Materials and Methods

### 2.1. Cell Culturing and Harvesting

The flagellate *Isochrysis galbana* (code CCMP1323) was cultured in Guillard’s F/2 medium without silicates. Experimental culturing was performed in triplicate in 10-liter polycarbonate bottles, with constant bubbling with air filtered through an 0.2 μm membrane filter in a climate chamber at 19 °C on a 12:12 h light:dark cycle and at 100 μmol photons m^−2^ s^−1^. Initial cell concentrations were approximately 5000 cells·mL^−1^ for each replicate, and culture growth was monitored daily from samples fixed with one drop of Lugol (final concentration of about 2%) and counted in a Bürker counting chamber under an Axioskop 2 microscope (20×) (Carl Zeiss GmbH) (as in Orefice et al. [33]). Aliquots of 50 mL were sampled in the exponential phase (6 days) and in the stationary phase (12 Days) and centrifuged for 15 min at 4 °C at 1900 *g* (Eppendorf, 5810 R). For RNA extraction, microalgal pellets were resuspended in 500 μL of TRIZOL© (Thermo Fisher Scientific, Waltham, MA, USA), incubated for 2–3 min at 60 °C until completely dissolved, frozen in liquid nitrogen and kept at −80 °C until the RNA extraction procedure (as in Lauritano et al. [34]). For chemical extraction and antiproliferative activity, pellets were frozen in liquid nitrogen and kept at −80 °C till further processing. 

### 2.2. RNA Extraction

RNA extraction was performed following the TRIZOL^®^ (Thermo Fisher Scientific, Waltham, MA, USA) manufacturer’s instructions, and quantity and quality were assessed as in Lauritano et al. [34]. Briefly, RNA quantity and quality were measured by using Nano-Drop (ND-1000 UV–Vis spectrophotometer; NanoDrop Technologies), monitoring the absorbance at 260 nm and the 260/280 nm and 260/230 nm ratios, respectively. RNA samples were considered good when both ratios were approximately 2. RNA quality was also assessed on 1% agarose gel and by measuring the RNA integrity number (RIN) with Agilent 2100 Bioanalyzer (Agilent Technologies, Inc., Santa Clara, CA, USA). RNA with RIN > 8 was considered to be of high quality and further processed.

### 2.3. RNA Sequencing, Transcriptome Assembly, Annotation, Expression Quantification and Differential Expression Analysis

Next-generation sequencing experiments were performed by Genomix4life S.R.L. (Baronissi, Salerno, Italy). RNA concentration in each sample was assayed with a NanoDrop ONE (Thermo Scientific), and its quality was assessed with the TapeStation 4200 (Agilent Technologies). Indexed libraries were prepared from 500 ng·ea^−1^ purified RNA TruSeq Stranded mRNA Sample Prep Kit (Illumina) according to the manufacturer’s instructions. Libraries were quantified using the TapeStation 4200 (Agilent Technologies) and Qubit fluorometer (Invitrogen Co., Waltham, MA, USA), then pooled such that each index-tagged sample was present in equimolar amounts, with a final concentration of the pooled samples of 2 nM. The pooled samples were subject to cluster generation and sequencing using an Illumina NextSeq 550Dx System (Illumina, San Diego, CA, USA) in a 2 × 75 paired-end format. Reads were processed to produce the transcriptome assembly as in [35]. Reads are freely available under the series entry PRJNA771997 in the NCBI BioProject database.

Fastq files were subjected to quality control and removal of adapter sequences using BBDuk [36]. The trimming step was performed with the following parameters: the minimum length was set to 35 bp and the quality score to 25. The software BBDuk was used for this scope. The quality of the reads was checked before and after the trimming step. The trimmed Illumina reads were used with the software Trinity [37] to generate a de novo assembly. A consensus assembly was obtained by first removing redundancy from each sample with the software CD-HIT-EST [38], then all the transcripts were joined, and a second round of CD-HIT-EST [38] was used to remove duplicated sequences. The completeness of the transcriptome assembly was then evaluated using the software BUSCO [39] against the databases of eukaryote conserved genes. The TransDecoder [40] pipeline was used to predict ORFs and protein sequences. The functional annotation of proteins was obtained using the PANNZER2 web server [41]. The expression of the predicted transcripts was estimated using the software Kallisto [42] with the RNA-seq reads. The obtained expression matrix was then analyzed with R, with the packages HTSFilter [43] and NOISeq [44,45] in order to identify differentially expressed transcripts between the two experimental groups.

### 2.4. Chemical Extraction and Pre-Fractionation

The wet microalgal samples were extracted by soaking in methanol and 30 min of maceration, vortexed for 1 min, sonicated with three bursts of 30 s in an icy water bath, and centrifuged at 3000 rpm and 4 °C (to precipitate the solid material in suspension, which was then discarded; as in Martinez et al. 2022 [46]). Samples were then transferred to a rounded Pyrex flask and evaporated under reduced pressure. An aliquot of methanolic extract with a maximum weight of 20 mg was fractionated by using CHROMABOND^®^ HR-X cartridges (6 mL·500 mg^−1^) as reported in Cutignano et al. [31]. Each fraction was then weighed and preserved at −20 °C.

### 2.5. In Vitro Antiproliferative Assay

Human cells were bought at ATCC (https://www.lgcstandards-atcc.org/, accessed on 1 June 2019). Human hepatocellular liver carcinoma (HepG2; ATCC^®^ HB-8065™) and human normal lung fibroblasts (MRC5; ATCC^®^ CCL-171™) were cultured in EMEM medium; human melanoma cells (A2058; ATCC^®^CRL-11147TM) were cultured in DMEM; adenocarcinomic human alveolar basal epithelial cells (A549; ATCC^®^CL-185TM) were cultured in F-12K medium; normal human keratinocytes (HaCat) were cultured in DMEM/F12 medium. The media were supplemented with 10% fetal bovine serum, 50 U·mL^−1^ penicillin and 50 μg·mL^−1^ streptomycin (media and supplement were from Thermo Fisher Scientific, Waltham, MA, USA). For the cell viability assay, cells were seeded in 96-well microtiter plates at a density of 1 × 10^4^ cells/well and incubated at 37 °C to allow for cell adhesion in each plate. After 24 h, the medium was replaced with fresh medium containing increasing concentrations of the total extracts or fractions (1, 10 and 100 μg·mL^−1^) and further incubated for 72 h. Each concentration was tested at least in triplicate. Cell viability was assessed using the 3-(4,5-dimethyl-2-thizolyl)-2,5-diphenyl-2H-tetrazolium bromide (MTT) test (A2231,0001, Applichem Pancreac Tischkalender, Darmstadt, GmbH). Briefly, the medium was replaced with medium containing MTT at 0.5 mg·mL^−1^, and the plates were incubated for 3 h at 37 °C. After incubation, cells were dissolved using isopropyl alcohol (used as MTT solvent). Absorbance was measured at OD = 570 nm using a microplate reader (Multiskan^TM^ FC Microplate Photometer, Thermo Fisher Scientific, Waltham, MA, USA) as in Riccio et al., 2021 [47]. Arithmetic means ± the standard deviations (SDs) were calculated and compared using a two-tailed Student’s *t* test. Differences at *p* < 0.05 were regarded as significant.

## 3. Results

### 3.1. Transcriptome Sequencing and De Novo Assembly

An RNA sequencing (RNA-seq) experiment was performed on six samples: a triplicate derived from the microalga *Isochrysis galbana* collected in the exponential phase (6 days) and a triplicate in the stationary phase (12 days). RNA-seq reads were assembled with the de novo approach. The starting dataset included Illumina reads from the six samples. Prior to further analysis, a quality check was performed on the raw Illumina sequencing data, removing low-quality portions while preserving the longest high-quality part of NGS reads. The obtained final transcriptome assembly statistics are reported in Table S1. The assembly was composed of 107,616 sequences ranging from 184 to 33,195 bp in length and with an N50 of 2003 bp. The mean GC content was 64.09%. The analysis of the completeness of the transcriptome assembly, assessed by BUSCO software based on databases of eukaryote conserved genes (ubiquitous and single-copy within phylogenetic lineages), showed a completeness of 74.9% (Figure S1).

### 3.2. Functional Annotation

Once the final assembly was produced, a transcriptome annotation was performed in order to identify coding sequences and to assign functions. The TransDecoder pipeline was used to predict ORFs and protein sequences. This approach led to the identification of 45,549 putative proteins, which were then functionally annotated with the PANNZER2 pipeline. The functional annotation led to the assignment of a description to 29,804 proteins and a gene ontology annotation to 25,074 sequences.

### 3.3. Differential Expression Analysis

There were 30,599 significantly differentially expressed genes. Considering only |LogFC| > 2, there were 9288 upregulated (among these, 2080 had an NCBI NR assignment) and 2086 downregulated genes (among these, 1244 had an NCBI NR assignment; reported in Table S2). The 10 most upregulated genes (6 days versus 12 days, used as control) were PPM-type phosphatase domain–containing protein, cyclic nucleotide-binding domain–containing protein, ankyrin repeat domain–containing protein 7, cytochrome b5 heme-binding domain–containing protein (fragment), outer dynein arm–docking complex subunit 3, cyclic nucleotide-binding domain–containing protein (fragment), chitin-binding type-4 domain–containing protein (fragment), prolyl 4-hydroxylase 4, trichohyalin-like and putative peroxisomal membrane protein (Table S2). On the contrary, between the most downregulated genes, there were PAS domain–containing protein (fragment), protein transport protein SEC24, eIF-2B GDP-GTP exchange factor subunit epsilon, hint domain–containing protein (fragment), succinate dehydrogenase [ubiquinone] flavoprotein subunit, mitochondrial, protein kinase domain–containing protein (fragment), C3H1-type domain–containing protein (fragment) and rRNA-processing arch domain–containing protein (Table S2).

For the significantly differentially expressed transcripts, a gene ontology enrichment analysis (GOEA) was performed to identify the most enriched gene ontology (GO) categories across the up- and downregulated sequences at 6 days compared to 12 days (Table 1 and Table 2 report up- and downregulated transcripts, respectively). The overall number of upregulated GO categories in biological processes (BPs), cellular component (CC) and molecular function (MF) were 99, 96 and 94, respectively. Regarding the downregulated GO categories, there were 79, 83 and 81 for BP, CC and MF, respectively. For the upregulated sequences at 6 days, the most represented categories were cellular protein complex disassembly, sterol biosynthetic process and regulation of protein localization for BP; recycling endosome membrane and proton-transporting V-type ATPase complex for CC; and protein tag, ADP-ribose diphosphatase activity and chorismate mutase activity for MF (Table 1). For the downregulated categories at 6 days, the most represented were photosystem II stabilization and NADH metabolic process for BP; myelin sheath, CORVET complex and TAT protein transport complex for CC; and D-cysteine desulfhydrase activity, sodium channel regulator activity and phosphodiesterase I activity for MF (Table 2).

### 3.4. Bioactivity and Chemical Fractionation of I. galbana Extract

**Figure 1:**
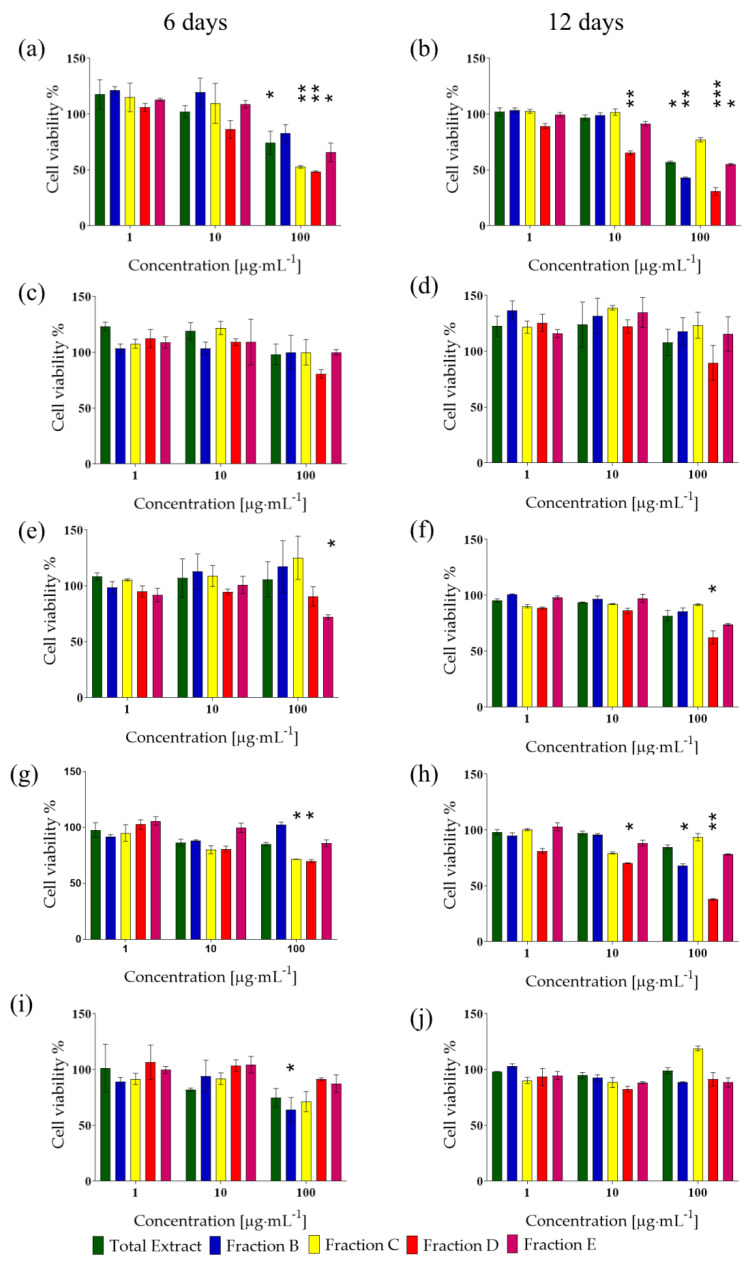
Cell viability assay. Antiproliferative effects of TE (green bars) or fractions (B blue bars; C yellow bars; D red bars; E purple bars) of *Isochrysis galbana* cultured for 6 days or 12 days on A2058 (**a**,**b**), HaCat (**c**,**d**), A549 (**e**,**f**), MRC5 (**g**,**h**) and HepG2 (**i**,**j**). Cell viability was normalized using cells incubated with complete cell medium in the presence of DMSO as a control sample. Results are expressed as percent survival after 72 h exposure (n = 3; * for *p* < 0,05; ** for *p* < 0.01 and *** for *p* < 0.001, Student’s *t*-test).

*I. galbana* (CCMP1323) methanolic extract (total extract, TE) was obtained from 10 L of *I. galbana* triplicate cultures collected at both the exponential growth phase (6 days culture) and stationary growth phase (12 days culture). In addition, enriched fractions (B-E) were obtained by solid-phase extraction (SPE) fractionation. Fraction A, obtained by this protocol, was discarded because it is rich in salts. Different concentrations (1–100 μg·mL^−1^) of total extracts and their enriched fractions (B–E) were tested for their potential to affect the viability of the human cancer cell lines, A2058 (melanoma cells), A549 (lung cancer cells) and HepG2 (hepatocarcinoma cells), HaCat (human keratinocytes) and normal human lung fibroblast MRC5. Total extracts of both 6- and 12-day pellets were selectively active at 100 µg·mL^−1^ only against A2058 cells (*p* < 0.05 for both, Figure 1a,b), while they did not show any antiproliferative activity on the other cancer cells or the normal cells, indicating the absence of a generic cytotoxicity.

Regarding fractions, for samples deriving from the exponential growth phase (6 days), major activity was found against melanoma A2058 cells. In particular, fractions C, D and E at 100 μg·mL^−1^ were able to reduce cell viability to 52%, 48% and 65% (*p* < 0.01 for fraction D and E, while *p* < 0.05 for fraction E; Figure 1a). Fraction E was slightly active at 100 μg·mL^−1^ against A549 cells also, reducing cell viability to 72% (*p* < 0.05; Figure 1b). Fraction B was active only on HepG2 cells, reducing cell viability to 64% when tested at 100 μg·mL^−1^ (*p* < 0.05; Figure 1i). Both fraction C and D at 100 μg·mL^−1^ also reduced the cell viability of MRC5 cells to 71% and 70%, respectively (*p* < 0.05 for both; Figure 1g).

As for the stationary phase, Fraction B and E reduced melanoma cell viability to 43% and 55%, respectively (*p* < 0.01 for fraction B and *p* < 0.05 for fraction E, Figure 1b). Fraction D was active against melanoma cells at both 10 and 100 µg·mL^−1^, reducing cell viability to 65% and 30%, respectively (*p* < 0.01 and *p* < 0.001, respectively; Figure 1b). Fraction D reduced A549 cells’ viability to 62% at 100 µg·mL^−1^ (*p* < 0.05; Figure 1f), did not affect HepG2 cells (*p* > 0.05; Figure 1j) and reduced MRC5 viability to 70% and 38%, when tested at 10 and 100 µg·mL^−1^ (*p* < 0.05 and *p* < 0.01, respectively; Figure 1h).

Finally, the total extract and the enriched fractions derived from both exponential growth phase and stationary growth phase did not show any significant cytotoxicity against normal human keratinocytes (HaCat, Figure 1c,d).

According to Cutignano et al. [31], fraction B is a nucleoside-enriched fraction, fraction C is a glycolipid and phospholipid-enriched fraction and fraction D is a sterol and free fatty acid–enriched fraction, while fraction E is a triglyceride-enriched fraction. Overall, our results showed that fractions B and E belonging to exponential growth phase samples (6 days) were the only fractions exerting activity on human cancer cells (fraction B on liver cells and fraction E on lung cells, respectively) without affecting normal cells. Regarding fractions derived from the microalga harvested in the stationary phase (12 days), fraction E was the only one with antiproliferative activity (on melanoma cells) with no effects on normal cells. To better clarify also at the gene level which metabolic pathways were highly expressed in *I. galbana* in the active conditions, transcriptome mining was performed.

### 3.5. Identification of Differentially Expressed Transcripts Related to Nucleoside, Glycolipid/Phospholipid, Sterol and Triglyceride Metabolism

Regarding the metabolism of nucleosides, nine transcripts coding Nudix (nucleoside diphosphate linked moiety X)-type motif 9, known to be involved in the hydrolysis of nucleoside diphosphate derivatives (https://www.genecards.org/cgi-bin/carddisp.pl?gene=NUDT9, accessed on 24 November 2021), were identified between the DEGs. The nine transcripts were upregulated at 6 days, with upregulation folds between 2.17 and 3.65. A transcript coding a putative peroxisomal adenine nucleotide transporter 1 was upregulated by 8.61 fold and another corresponding to the Alsin Rho guanine nucleotide exchange factor ALS2 b by 2.36 fold. A transcript coding deoxynucleoside triphosphate triphosphohydrolase SAMHD1, a protein known to be involved in the defense response to viruses and as a regulator of DNA end resection at stalled replication forks (https://www.uniprot.org/uniprot/Q9Y3Z3, accessed on 24 November 2021), was downregulated by −2.13 fold; two transcripts for ribonucleoside-diphosphate reductase, which catalyze the formation of deoxyribonucleotides from ribonucleotides [48], were downregulated by −2.14 and −8.36 fold, as well as a nucleoside hydrolase of −2.21 fold, a guanine deaminase of −2.79 fold and a purine nucleoside permease (involved in adenosine and guanosine transport; https://www.uniprot.org/uniprot/Q5AGW8, accessed on 28 March 2022) by −2.90 fold.

Interestingly, various transcripts were identified in DEGs as being involved in nucleotide methyltransferase activity, suggesting methylation variation (possible epigenetic regulation), such as a tRNA (cytosine(34)-C(5))-methyltransferase mitochondrial, which was downregulated by −2.10 fold; a putative tRNA/rRNA cytosine-C5-methylase by −2.84 fold; a site-specific DNA-methyltransferase (cytosine-N(4)-specific) by −4.11 fold and a RNA (guanine-9-)-methyltransferase domain-containing protein 2 by −5.81 fold. Two transcripts were identified as coding a DNA (cytosine-5)-methyltransferase CMT1, but one was 6.31-fold upregulated, while the other was −2.25-fold downregulated.

Regarding the metabolism of glycolipids and phospholipids, mainly transporters were differentially expressed. Four transcripts coding for phospholipid-transporting ATPase were identified as upregulated at 6 days, with an upregulation between 2.09 to 2.55 fold. Two transcripts coding for phospholipid-transporting ATPase ABCA1 were upregulated at 6 days by 2.51 and 3.12 fold. A transcript coding for P-type phospholipid transporter was downregulated at 6 days by −2.08 fold. Seven transcripts coding phosphatidylcholine-sterol O-acyltransferase (EC number 2.3.1.43) were upregulated at 6 days by 3.09, 2.77, 2.62, 2.61, 2.55, 2.42 and 2.20 fold, respectively. These enzymes catalyze the synthesis of 1-acyl-sn-glycero-3-phosphocholine using phosphatidylcholine as a substrate (KEGG Kyoto Encyclopedia of Genes and Genomes https://www.genome.jp/pathway/ehx00564+EMIHUDRAFT_434784, accessed on 15 November 2021). A transcript annotated as CDP-diacylglycerol synthase (EC number 2.7.7.41) was downregulated at 6 days by −3.51 fold. Two transcripts annotated as glycerophosphocholine acyltransferase 1 (EC number 2.3.1.23) were downregulated at 6 days by −3.01 and −2.35 fold, respectively. Two transcripts annotated as diacylglycerol kinase (EC number 2.7.1.107) were downregulated at 6 days by −3.00 and −2.12 fold. A transcript coding sulfoquinovosyldiacylglycerol 2 (EC number 2.4.1.-) was −2.11-fold downregulated. A transcript annotated as S-acyltransferase (EC number 2.3.1.225) was −4,67-fold downregulated at 6 days.

Looking for all the annotated DEGs related to “sterol”, some transcripts were up-regulated at 6 days mainly indicating an increase in the synthesis of cholesterol. In particular, three transcripts coding for delta(24)-sterol reductase (EC 1.3.1.72), participating in the biosynthesis of steroids in different points of the biosynthetic pathway, were upregulated by 2.96, 2.66 and 2.25 fold, respectively, a transcript coding for sterol 14-demethylase (EC 1.14.14.154) was 2.94-fold upregulated; six transcripts coding for 7-dehydrocholesterol reductase (EC number 1.3.1.21), involved in the production of cholesterol from 7-dehydrocholesterol, were upregulated by 3.67, 3.26, 2.99, 2.97, 2.94 and 2.26 fold, respectively, and the ERG4/ERG24 ergosterol biosynthesis family protein showed a 2.78-fold upregulation.

Moreover, a transcript annotated as a terpene cyclase/mutase (upregulated 2.39 fold) family member was found to be related to cycloartenol synthase (EC 5.4.99.8) (megablastp on the NCBI server https://blast.ncbi.nlm.nih.gov/Blast.cgi, accessed on 13 October 2021), an enzyme involved in the synthesis of phytosterol precursors.

According to [49], triglycerides may be synthesized either via the glycerol phosphate/Kennedy pathway or the monoacylglycerol (MG) pathway. Looking for enzymes involved in these pathways, we only found glycerol-phosphate acyltransferase, which was downregulated by −2.18 fold at 6 days. Considering that for both 6 days and 12 days, the triglyceride-enriched fraction was the most active, we did not expect variations for the biosynthetic pathways. In fact, the diacylglycerol acyltransferase (DGAT) enzyme, known to convert a fatty acyl-CoA and diacylglycerol molecule to form triglycerides [49], was not differentially expressed.

### 3.6. Differentially Expressed Genes Related to the Synthesis of Secondary Metabolites with Known Anticancer Activity

Microalgal terpenoids, e.g., carotenoids, have been reported to exert anticancer activity against various cancer cells [50,51]. Transcripts related to terpenoid backbone biosynthesis were found to be upregulated at 6 days. Several transcripts were related to the mevalonate pathway. In particular, a transcript annotated as mevalonate kinase (EC number 2.7.1.36) was 2.86-fold upregulated; two transcripts annotated as phosphomevalonate kinase (EC number 2.7.4.2) were 2.19- and 2.09-fold upregulated; and seven transcripts annotated as 3-hydroxy-3-methylglutaryl-coenzyme A synthase 1 (EC number 2.3.3.10) were 3.59-, 2.80-, 2.77-, 2.64-, 2.58-, 2.37- and 2.16-fold upregulated, respectively. A transcript related to methylerythritol phosphate (MEP), annotated as 4-diphosphocytidyl-2C-methyl-D-erythritol synthase (EC number 2.7.7.60), was 8.79-fold upregulated. A transcript coding for an isopentenyl-diphosphate delta-isomerase (EC number 5.3.3.2) was 2.07-fold upregulated. Seven transcripts annotated as farnesyl pyrophosphate synthase (EC number 2.5.1.10) were 3.85-, 3.29-, 2.96-, 2.92-, 2.30-, 2.20- and 2.04- fold upregulated. A transcript coding for carotene isomerase was −3.89-fold downregulated, while two transcripts annotated as 9,9’-di-cis-ζ-carotene desaturase (EC number 1.3.5.6) were 2.38- and 2.11-fold upregulated.

In recent studies, prostaglandins, commonly associated with inflammation, have been suggested to also be involved in cancer [52]. Interestingly, two transcripts related to prostaglandin biosynthesis were found between DEGs. A transcript coding for a microsomal prostaglandin E synthase 2 (Fragment) was −2.42-fold downregulated, and a transcript coding for a prostaglandin F synthase was −3.17-fold downregulated.

## 4. Discussion

*Isochrysis* spp. were previously shown to produce valuable compounds, such as carotenoids, lipids and carbohydrates, with possible useful bioactivities for human health, including antioxidant, antimicrobial and antiproliferative (against human lymphoblast) activities and for aquaculture [15,16,17,18,53,54,55,56]. As reviewed by Ramos-Romero and colleagues [57], *I. galbana* is also among the edible microalgae with activities useful for the prevention and treatment of metabolic alterations. Recent studies have suggested *I. galbana* as ingredient for functional foods due to its richness in omega3 long-chain polyunsaturated fatty acids, and oleic, linoleic, alpha-linolenic acid, stearidonic and docosahexaenoic acids [53]. As summarized by Ferreira et al. [58], *I. galbana* has been used for the preparation of bread, chikki, cookies, crackers, pasta, noodles, tomato puree, burgers, beef patties and chewing gum. The genome of *I. galbana* was also recently sequenced using PacBio RSII sequencing technology (BioProject number PRJNA669236), providing a high-quality genome assembly (assembly accession number GCA_018136815.1) with 15 total chromosomes. In the current study, we performed a de novo transcriptome assembly of *I. galbana* and identified the annotated and differentially expressed genes various transcripts related to nucleoside, phospholipid, glycolipid and sterol metabolism.

We also screened both total extracts and fractions from *I. galbana* cultures harvested in the exponential or stationary phase against a panel of cancer cells, including different tissues (i.e., skin, lung and liver) and found a selective antiproliferative activity of total extracts against melanoma A2058 cells. Bioactivity-guided fractionation allowed the identification, as the most active, of fractions B and E belonging to exponential growth phase samples (6 days) and fraction E from the stationary phase (12 days), with no effects on normal cells. Fractions C and D from the 6-day samples were also active on melanoma and normal cells, fraction B from the 12-day samples was active on melanoma and normal cells, while fraction D from the 12-day samples was active on melanoma, lung and normal cells.

By analyzing the differentially expressed transcripts, we did not observe a differential expression for triglyceride-metabolism-related transcripts, suggesting that this pathway did not vary in the two growth phases studied. This is in line with the conserved bioactivity observed for fraction E for both 6- and 12-day conditions.

Regarding transcripts involved in nucleoside metabolism, the major part of the DEGs was downregulated at 6 days (compared to 12 days), such as two transcripts coding for ribonucleoside-diphosphate reductase, which catalyzes the formation of deoxyribonucleotides from ribonucleotides [48]; a deoxynucleoside triphosphate triphosphohydrolase SAMHD1, known to convert deoxynucleoside triphosphates to deoxynucleosides [59]; a nucleoside hydrolase and a purine nucleoside permease, which transports adenosine and guanosine (https://www.ebi.ac.uk/interpro/entry/InterPro/IPR009486/; accessed on 21 September 2021).

Regarding DEGs related to phospholipid metabolism, there was the upregulation of a transcript coding for phosphoinositide phospholipase C, known to be involved in lipid signaling pathways [60], a transcript coding for lysophospholipase/carboxylesterase family protein and six transcripts coding for phospholipid-transporting ATPase (Table S2). Another transcript related to transport activities, annotated as P-type phospholipid transporter was downregulated, and a transcript annotated as phospholipase, involved in the hydrolysis of phospholipids into fatty acids and other lipophilic substances, was downregulated (Table S2). Transporters known as adenosine triphosphate (ATP)–binding cassette (ABC) super family transporters are energy-dependent protein pumps that act as active efflux pumps, often studied/known because they lead to lower intracellular accumulation of xenobiotic substrates. However, studies have shown that this transporter family comprises several members with different affinities and specificities. For instance, in the human genome, 48 ABC transporters classified into seven subfamilies have been reported [61]. Our results suggest the activation and deactivation of specific transporters indicating a timely regulated system to increase the accumulation of specific compounds. Regarding glycolipid metabolism, we did find the upregulation at 6 days of seven transcripts annotated as phosphatidylcholine-sterol O-acyltransferase (EC 2.3.1.43), known to covert a phosphatidylcholine and a sterol into 1-acylglycerophosphocholine and a sterol ester, and one transcript annotated as sterol 3-beta-glucosyltransferase, involved in the biosynthesis of sterol glucosides (https://www.uniprot.org/uniprot/Q9XIG1; accessed on 21 September 2021).

## 5. Conclusions

Overall, these findings provide additional evidence on the role of microalgae as a source of high-value compounds. In fact, *I. galbana* has antiproliferative activity against melanoma and lung carcinoma, in addition to the previously identified activity on lung lymphoblasts, suggesting new potential applications in the cancer sector. The approach used in the current study, combining transcriptome analyses and bioactivity testing, allowed the identification of key enzymes, which may be involved in the synthesis of metabolites with antiproliferative activity as well as possible target sequences for future gene-editing studies. Gene manipulation may direct algal metabolism for the production of high yields of the metabolites of interest. Overall, these data show how the transcriptomic approach is a powerful tool to assist and guide drug discovery from marine microalgae, showing timely regulated pathways responsible for the synthesis of bioactive groups of metabolites. The performed sequencing renders available new molecular resources for marine microalgae, still poorly explored for their bioactivity compared to terrestrial species, which can be precious for ecological as well as biotechnological and bioengineering studies.

## Figures and Tables

**Table 1:** The top ten gene ontology (GO) categories in biological processes (BPs), cellular component (CC) and molecular function (MF) in the upregulated group of transcripts at 6 days.

GO Class	GO Description	FDR	Enrichment Score
BP	cellular protein complex disassembly	3.78 × 10^−9^	3.54
BP	sterol biosynthetic process	3.03 × 10^−9^	3.47
BP	regulation of protein localization	1.42 × 10^−7^	3.30
BP	protein localization to membrane	3.28 × 10^−9^	3.21
BP	positive regulation of RNA polymerase II transcriptional preinitiation complex assembly	1.87 × 10^−4^	3.10
BP	nuclear-transcribed mRNA catabolic process, deadenylation-dependent decay	2.73 × 10^−5^	3.07
BP	negative regulation of DNA recombination	1.30 × 10^−4^	3.03
BP	acetyl-CoA metabolic process	1.31 × 10^−3^	2.79
BP	organic cyclic compound biosynthetic process	8.64 × 10^−4^	2.75
BP	carbohydrate derivative biosynthetic process	5.61 × 10^−4^	2.72
CC	recycling endosome membrane	3.12 × 10^−6^	4.40
CC	proton-transporting V-type ATPase complex	5.60 × 10^−6^	4.05
CC	AP-2 adaptor complex	4.56 × 10^−4^	3.72
CC	WASH complex	1.81 × 10^−3^	3.54
CC	eukaryotic translation initiation factor 2 complex	1.81 × 10^−3^	3.54
CC	transporter complex	4.27 × 10^−4^	3.47
CC	clathrin coat of coated pit	1.99 × 10^−5^	3.41
CC	endocytic vesicle	1.54 × 10^−5^	3.30
CC	clathrin coat of trans-Golgi network vesicle	7.34 × 10^−5^	3.30
CC	proton-transporting ATP synthase complex, catalytic core F(1)	7.34 × 10^−5^	3.30
MF	protein tag	5.60 × 10^−6^	4.05
MF	ADP-ribose diphosphatase activity	1.23 × 10^−9^	4.01
MF	chorismate mutase activity	2.69 × 10^−5^	3.72
MF	structural constituent of cell wall	5.33 × 10^−6^	3.50
MF	clathrin adaptor activity	9.37 × 10^−5^	3.43
MF	oxidoreductase activity, acting on single donors with incorporation of molecular oxygen, incorporation of two atoms of oxygen	1.92 × 10^−12^	3.27
MF	beta-tubulin binding	1.24 × 10^−3^	3.15
MF	ATPase activator activity	3.98 × 10^−6^	3.05
MF	clathrin light chain binding	6.16 × 10^−4^	2.97
MF	ubiquitin conjugating enzyme activity	6.16 × 10^−4^	2.97

**Table 2:** The top ten enriched gene ontology (GO) categories in molecular function (MF), cellular component (CC) and biological processes (BPs) in the downregulated group of transcripts at 6 days.

GO Class	GO Description	FDR	Enrichment Score
BP	photosystem II stabilization	3.09 × 10^−6^	3.02
BP	NADH metabolic process	3.35 × 10^−5^	2.97
BP	response to high light intensity	1.10 × 10^−4^	2.93
BP	protein localization to nucleolar rDNA repeats	1.10 × 10^−4^	2.93
BP	7-methylguanosine mRNA capping	1.10 × 10^−4^	2.93
BP	lipid A biosynthetic process	4.08 × 10^−4^	2.53
BP	photosynthesis, light harvesting	0.00 × 10	2.41
BP	heterochromatin organization	3.09 × 10^−3^	2.40
BP	glycerophospholipid metabolic process	8.20 × 10^−3^	2.31
BP	cellular ketone metabolic process	8.20 × 10^−3^	2.31
CC	myelin sheath	0.00 × 10	3.30
CC	CORVET complex	0.00 × 10	3.30
CC	TAT protein transport complex	0.00 × 10	3.30
CC	phosphatidylinositol 3-kinase complex, class III	0.00 × 10	3.30
CC	ribonuclease MRP complex	0.00 × 10	3.30
CC	mRNA cap binding complex	0.00 × 10	3.30
CC	contractile actin filament bundle	0.00 × 10	3.30
CC	Chromocenter	0.00 × 10	3.30
CC	glutathione synthase complex	0.00 × 10	3.30
CC	sarcoplasmic reticulum	0.00 × 10	3.30
MF	D-cysteine desulfhydrase activity	0.00 × 10	3.30
MF	sodium channel regulator activity	0.00 × 10	3.30
MF	phosphodiesterase I activity	0.00 × 10	3.30
MF	isocitrate dehydrogenase (NADP+) activity	1.17 × 10^−3^	2.82
MF	glutathione-disulfide reductase activity	1.17 × 10^−3^	2.82
MF	mRNA guanylyltransferase activity	3.77 × 10^−3^	2.75
MF	palmitoyl-(protein) hydrolase activity	1.17 × 10^−2^	2.64
MF	alpha-L-arabinofuranosidase activity	1.17 × 10^−2^	2.64
MF	pyruvate carboxylase activity	1.17 × 10^−2^	2.64
MF	plastoquinol–plastocyanin reductase activity	1.17 × 10^−2^	2.64

## Data Availability

*Isochrysis galbana* transcriptome reads are freely available under the series entry PRJNA771997 in the NCBI BioProject database.

## Supplementary Materials

The following are available online at https://www.mdpi.com/article/10.3390/biology11050771/s1: Figure S1: Graphical representation of the results of the BUSCO analysis using the eukaryote databases. C = complete; S = single copy; D = duplicated; F = fragmented; M = missing. Table S1: *Isochrysis galbana* transcriptome assembly statistics. Table S2: Differentially expressed genes.
