# Supplementary material for: De Novo Transcriptome of the Flagellate Isochrysis galbana Identifies Genes Involved in the Metabolism of Antiproliferative Metabolites
Source: Biology (Basel). 2022 May 18;11(5):771. doi: 10.3390/biology11050771 (PMC9138222; doi:10.3390/biology11050771)

Figure S1. Graphical representation of the results of the BUSCO analysis using the Eukaryote databases. C=complete; S=single copy; D=duplicated; F=fragmented; M=missing.

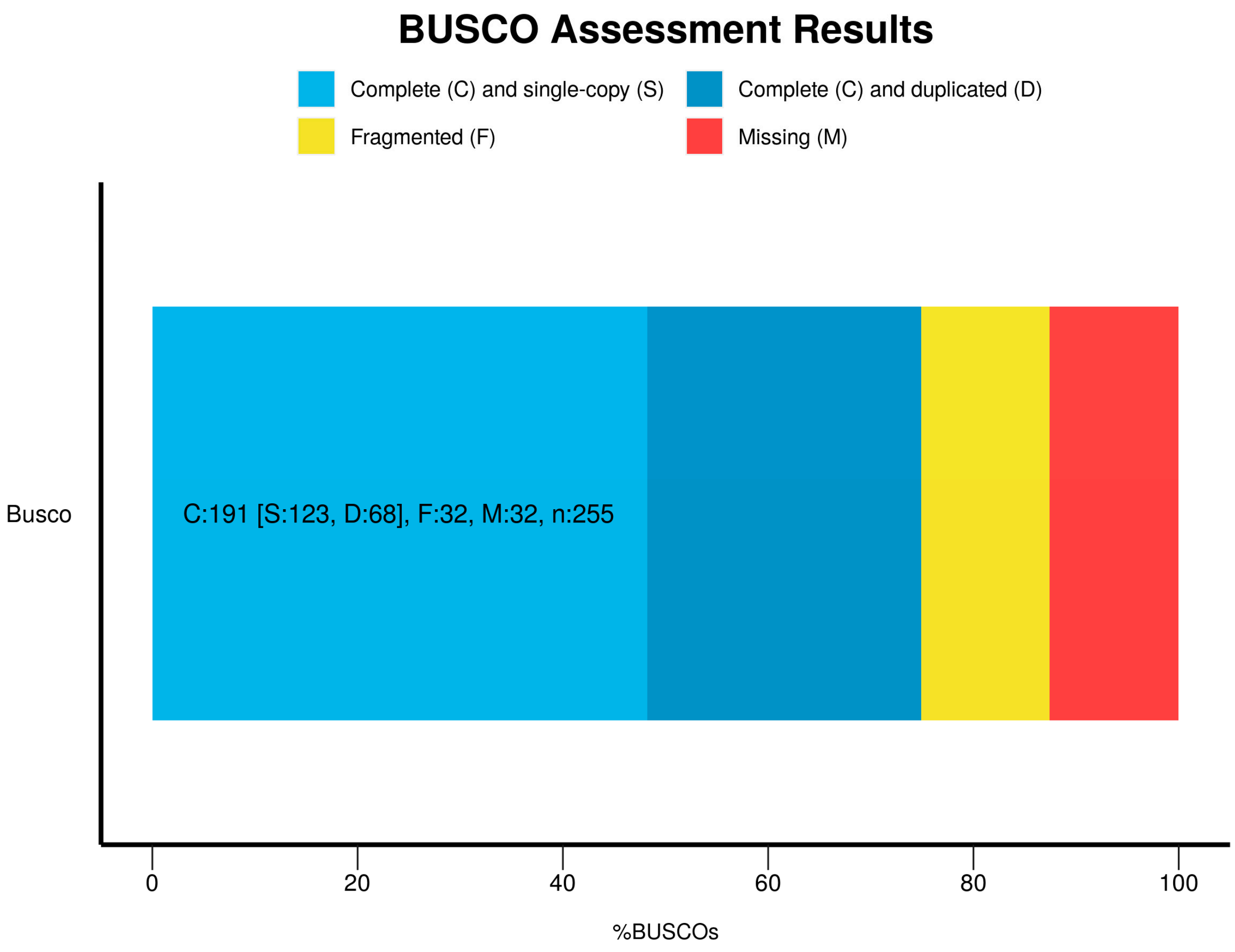

Supplement: Supplementary file 1 [file biology-11-00771-s001.zip › Figure S1.pdf]
