# Supplementary material for: De Novo Transcriptome of the Flagellate Isochrysis galbana Identifies Genes Involved in the Metabolism of Antiproliferative Metabolites
Source: Biology (Basel). 2022 May 18;11(5):771. doi: 10.3390/biology11050771 (PMC9138222; doi:10.3390/biology11050771)
Supplement: Supplementary file 1 [file biology-11-00771-s001.zip › Table S1.pdf]

Table S1. *Isochrysis galbana* transcriptome assembly statistics

# contigs (>= 0 bp)	107616
# contigs (>= 1000 bp)	33095
# contigs (>= 5000 bp)	837
# contigs (>= 10000 bp)	79
# contigs (>= 25000 bp)	3
# contigs (>= 50000 bp)	0
Largest contig	33195
Total length	80409305
GC (%)	64.09
N50	2003
N75	1309
L50	13066
L75	25443
